# Childhood maltreatment and cognitive functioning: the role of depression, parental education, and polygenic predisposition

**DOI:** 10.1038/s41386-020-00794-6

**Published:** 2020-08-14

**Authors:** Janik Goltermann, Ronny Redlich, Dominik Grotegerd, Katharina Dohm, Elisabeth J. Leehr, Joscha Böhnlein, Katharina Förster, Susanne Meinert, Verena Enneking, Maike Richter, Jonathan Repple, Immanuel DeVillers, Marine Kloecker, Andreas Jansen, Axel Krug, Igor Nenadić, Katharina Brosch, Tina Meller, Frederike Stein, Simon Schmitt, Marcella Rietschel, Fabian Streit, Stephanie H. Witt, Andreas J. Forstner, Markus M. Nöthen, Bernhard T. Baune, Till F. M. Andlauer, Tilo Kircher, Nils Opel, Udo Dannlowski

**Affiliations:** 1 Department of Psychiatry, University of Münster, Münster, Germany; 2 Department of Psychiatry, University of Marburg, Marburg, Germany; 3 Department of Psychiatry and Psychotherapy, University of Bonn, Bonn, Germany; 4 Department of Genetic Epidemiology, Central Institute of Mental Health, University of Heidelberg, Mannheim, Germany; 5 Institute of Human Genetics, School of Medicine & University Hospital Bonn, University of Bonn, Bonn, Germany; 6 Department of Biomedicine, University of Basel, Basel, Switzerland; 7 Centre for Human Genetics, University of Marburg, Marburg, Germany; 8 Department of Psychiatry, Melbourne Medical School, The University of Melbourne, Melbourne, VIC Australia; 9 The Florey Institute of Neuroscience and Mental Health, The University of Melbourne, Parkville, VIC Australia; 10 Department of Neurology, Klinikum rechts der Isar, School of Medicine, Technical University of Munich, Munich, Germany

**Keywords:** Risk factors, Genetic interaction, Depression

## Abstract

Childhood maltreatment is associated with cognitive deficits that in turn have been predictive for therapeutic outcome in psychiatric patients. However, previous studies have either investigated maltreatment associations with single cognitive domains or failed to adequately control for confounders such as depression, socioeconomic environment, and genetic predisposition. We aimed to isolate the relationship between childhood maltreatment and dysfunction in diverse cognitive domains, while estimating the contribution of potential confounders to this relationship, and to investigate gene–environment interactions. We included 547 depressive disorder and 670 healthy control participants (mean age: 34.7 years, SD = 13.2). Cognitive functioning was assessed for the domains of working memory, executive functioning, processing speed, attention, memory, and verbal intelligence using neuropsychological tests. Childhood maltreatment and parental education were assessed using self-reports, and psychiatric diagnosis was based on DSM-IV criteria. Polygenic scores for depression and for educational attainment were calculated. Multivariate analysis of cognitive domains yielded significant associations with childhood maltreatment (*η*²_p_ = 0.083, *P* < 0.001), depression (*η*²_p_ = 0.097, *P* < 0.001), parental education (*η*²_p_ = 0.085, *P* < 0.001), and polygenic scores for depression (*η*²_p_ = 0.021, *P* = 0.005) and educational attainment (*η*²_p_ = 0.031, *P* < 0.001). Each of these associations remained significant when including all of the predictors in one model. Univariate tests revealed that maltreatment was associated with poorer performance in all cognitive domains. Thus, environmental, psychopathological, and genetic risk factors each independently affect cognition. The insights of the current study may aid in estimating the potential impact of different loci of interventions for cognitive dysfunction. Future research should investigate if customized interventions, informed by individual risk profiles and related cognitive preconditions, might enhance response to therapeutic treatments.

## Introduction

Childhood maltreatment (CM) has been discussed as a major vulnerability factor for various psychiatric disorders, including affective disorders, anxiety disorders, and posttraumatic stress disorder (PTSD) [1], as well as personality disorders [2]. Regarding major depressive disorder (MDD), there is growing evidence that CM is associated with higher risk to develop MDD, higher symptom severity, an unfavorable disease trajectory, and lower treatment response [3, 4]. While these findings stress the relevance of CM to psychiatric research, it is uncertain what mechanisms drive these adverse effects.

Research suggests that repeated or enduring stress during sensitive developmental stages of childhood or adolescence promotes biological long-term adverse effects, particularly in limbic and prefrontal areas, that persist until adulthood [5–9]. These neurobiological alterations are accompanied by functional deficits in various cognitive domains. While deteriorating effects of CM on working memory, attention, and intelligence have been reported consistently [10–12], there are mixed results regarding episodic memory, processing speed, and executive functioning [10, 11, 13]. Previous meta-analyses seem to disagree on whether CM associations with cognitive functioning are evident non-specifically across cognitive domains or differ across domains. One meta-analysis comparing healthy CM and non-CM groups found widespread cognitive deficits in the CM group irrespective of the cognitive domains [11]. Another meta-analysis investigating samples with several psychiatric diagnoses found that largest effects of CM on cognition were evident in the attention and visual episodic memory domains, while no associations were observed for visuospatial working memory and executive functioning [13]. On the contrary, Masson et al. found in their meta-analysis that the strongest associations between CM and cognition were evident in the executive functioning domain [14]. Thus, findings are inconsistent in regard to the cognitive domains where effects emerge. Generally, CM-related cognitive deficits can already be found in children, seem to persist until adulthood [11], and can be associated with CM in PTSD and non-PTSD samples [15]. Further, it is unclear to what extent the association between CM and cognitive functioning may be produced by common environmental and genetic factors.

Many of the CM-related cognitive deficits are also reported in MDD samples [16]. Grassi-Oliveira et al. found lower memory performance in a group of physically neglected MDD patients compared to MDD and healthy control (HC) groups without maltreatment experiences indicating that maltreatment has an adverse effect beyond psychiatric diagnosis [17]. However, despite an extensive literature investigating the link between CM and cognition, many studies do not control or screen for psychiatric comorbidities [12]. Further, there is evidence that points to a crucial role of cognitive dysfunction for the treatment of MDD as it (similar to CM) has been predictive for poorer treatment response in antidepressive pharmacotherapy and cognitive-behavioral psychotherapy [18]. Due to these interrelations, it is difficult to ascribe whether cognitive deficits are due to CM or merely reflect a higher percentage of MDD in maltreated samples.

Another variable that is associated with both CM and cognitive functioning is the familial socioeconomic status (SES) that defines a child’s environment, e.g., determined by family income, occupational status, or parental education (PE). Children from low SES families are at an increased risk to experience abuse and neglect compared to children from high SES families [19]. Further, low parental SES has been linked to cognitive deficits in various domains already at young age [19], and educational attainment (EdA) later in life [20]. Thus, familial SES may also confound the relationship between CM and cognitive abilities. However, ~41% of studies investigating CM-related cognitive dysfunction do not methodologically or statistically control for SES [15]. Previous meta-analytic analysis indicated that there is no crucial relevance of SES for the relationship between CM and cognition [11]. However, more recent work investigating two large prospective cohorts, reported that effect sizes of CM on cognition decreased considerably when controlling for SES [21]. The authors argue that there is no causal relationship between CM and cognition but that familial SES confounds this association.

It is further unclear if effects of familial SES on cognition are brought about purely by environmental disadvantages or might have a substantial genetic component. Data from 3000 genotyped children showed a common genetic influence on family SES and cognition [22]. Therefore, it is possible that a genetic predisposition might confound the relationship between CM and cognition.

Similarly, the suggested importance of MDD for the association between CM and cognition may be confounded by a genetic vulnerability for MDD. Familial liability for affective disorders has been associated with higher prevalence and more detrimental effects of CM (i.e., a gene–environment interaction) [23].

The aim of the current work is to disentangle the complex interplay between maltreatment, depression, familial SES, genetic predisposition, and cognitive abilities. Therefore, we examine the individual and combined influence of MDD diagnosis, PE, and genetic variables on the association between CM and cognitive dysfunction. Further, potential interaction effects between maltreatment and the above variables on cognition are investigated, including gene–environment interactions.

Based on the literature outlined above, we expect that CM, MDD diagnosis, and the genetic predisposition for MDD are all negatively associated with cognitive abilities, while higher PE and the genetic predisposition for EdA are both positively associated with cognitive performance. We further test the hypothesis that there is an independent effect of CM on cognition even when controlling for MDD diagnosis, PE and genetic variables. In addition to the hypothesis-based analyses, potential interaction effects of CM with age, sex, MDD diagnosis, PE, and genetic predisposition are explored.

## Methods

### Participants

For the present analyses all MDD and HC participants from the FOR2107 Marburg–Münster Affective Disorders Cohort Study (MACS) [24] with complete assessments of CM, psychiatric diagnosis, PE, genetics, and at least one neurocognitive measure were included. This resulted in a study sample of *n* = 547 MDD and *n* = 670 HC participants (total *N* = 1217, mean age: 34.7, SD = 13.2; 62.4% female). The MACS includes adults with age 18–65 years. Sample characteristics are displayed in Table 1.

**Table 1:** Study sample characteristics by diagnosis groups.

		MDD		HC		
	*N*	Mean	SD	Mean	SD	*p* value
Demographics						
Sex (f/m)	760/457	336/211		424/246		0.506
Age	1217	37.28	13.53	32.51	12.48	<0.001
PE	1217	11.95	2.89	13.25	3.09	<0.001
Maltreatment						
CTQ sum	1217	46.13	15.99	32.13	8.31	<0.001
CTQ EA	1217	11.26	5.22	6.96	2.86	<0.001
CTQ PA	1217	7.07	3.35	5.55	1.55	<0.001
CTQ SA	1217	6.35	3.19	5.22	1.15	<0.001
CTQ EN	1217	13.39	5.30	8.25	3.52	<0.001
CTQ PN	1217	8.07	3.21	6.15	1.79	<0.001
Cognitive measures						
Corsi A	1214	8.61	1.90	9.34	1.96	<0.001
Corsi B	1214	8.03	1.94	8.90	1.73	<0.001
LNST	1212	15.68	3.36	16.73	3.09	<0.001
TMT A–B	1210	−32.60	22.35	−25.55	16.38	<0.001
TMT A	1214	−26.36	10.24	−23.09	8.00	<0.001
DSST	1215	55.58	12.52	64.35	11.45	<0.001
d2	1211	166.59	43.64	191.04	42.85	<0.001
VLMT A	1215	55.23	10.32	59.41	8.56	<0.001
VLMT B	1214	13.02	3.10	13.83	1.95	<0.001
MWT-B	1214	112.75	13.76	114.06	13.57	0.097
Polygenic scores						
MDD PGS	1217	0.10	0.94	−0.09	1.03	0.001
EdA PGS	1217	−0.06	1.02	0.06	0.98	0.039
Depression severity						
HDRS	1212	9.85	7.55	1.43	2.13	<0.001

A *χ*²-test was used to test for significance of group differences regarding sex and *t* tests were used to test for significance of group differences in other variables. Statistics are presented assuming unequal variances in groups as most dependent variables produced a significant Levene’s test. Both PGS’ are presented *z*-standardized. For all cognitive measures, higher scores represent better performance.

*MDD* major depressive disorder, *HC* healthy controls, *f* female, *m* male, *CTQ* childhood trauma questionnaire, *EA* emotional abuse, *PA* physical abuse, *SA* sexual abuse, *EN* emotional neglect, *PN* physical neglect, *PE* parental education, *Corsi A* Corsi block tapping task forward, *Corsi B* Corsi block tapping task backward, *LNST* letter-number sequencing test, *TMT* trailmaking test (versions A and B), *DSST* digit symbol substitution test, *d2* d2 test, *VLMT* verbal learning and memory test (*VLMT A* short-term memory, *VLMT B* long-term memory), *MWT-B* multiple choice vocabulary test B, *PGS* polygenic score, *HDRS* Hamilton Depression Rating Scale.

All participants gave written and informed consent and received financial compensation. Exclusion criteria and participant recruitment are described in the Supplementary.

### Materials and procedure

#### Assessment of childhood maltreatment and parental education

The childhood trauma questionnaire (CTQ) was used to assess CM [25]—a retrospective self-report questionnaire with good psychometric qualities [25]. It allows the calculation of a CTQ sum score (indicating the general burden of maltreatment experiences during childhood) and five subtype scores.

PE of participants was used as a proxy for the socioeconomic environment during childhood and adolescence. PE was operationalized as the parental years of education assessed by self-report of the participants.

#### Neurocognitive assessment

A neurocognitive test battery was employed containing measures of working memory (Corsi A, Corsi B [26], and the letter-number sequencing test [LNST] [27]), executive functioning (trailmaking test [TMT] A–B [28]), processing speed (TMT A and digit symbol substitution test [DSST] [27]), sustained attention (d2 test [29]), declarative short-term memory (verbal learning and memory test [VLMT] A [30]), declarative long-term memory (VLMT B), and verbal IQ (multiple choice vocabulary test [MWT-B] [31]). The neurocognitive tests were applied in the following fixed order: VLMT A, TMT A, TMT B, Corsi A, Corsi B, LNST, DSST, d2 test, VLMT B, and MWT-B. See Supplementary for details on the operationalization of cognitive performance measures.

#### Clinical assessment

A structured clinical interview for DSM-IV (SCID-I) [32] was conducted with each participant in order to assess current and lifetime psychiatric diagnoses. Subjects from the MDD group fulfilled the DSM-IV criteria for either an acute or a lifetime history major depressive episode. It was secured that all HC subjects were free from any acute or history of psychiatric disorders based on the SCID-I. Current depression severity was additionally assessed using the Hamilton Depression Rating Scale [33].

#### Genetic assessment and quality control

A polygenic approach was applied in order to operationalize the genetic predispositions for MDD and EdA. The resulting polygenic scores (PGS) can be seen as the genetic propensity equivalents to the actual presence of an MDD diagnosis and PE.

To this end, DNA extraction and genome-wide genotyping, as well as quality control of the genotype data and the calculation of PGS were conducted as described elsewhere [34]. Quality-controlled single nucleotide polymorphisms (SNPs) were clumped in PLINK v1.9 [35] to create a set of linkage disequilibrium-independent variants, based on *p* values from published genome-wide association studies (GWAS). Clumping parameters were:—clump-p1 1—clump-p2 1—clump-r2 0.1—clump-kb 250. For each participant, PGS for MDD and EdA were generated using all available SNPs (threshold *P* = 1.0) from published large-scale MDD [36] and EdA [37] GWAS. Both GWAS samples are completely independent of the MACS sample used in this study. The PGS were calculated as the sum of independent SNPs (genotype dosage from 0 to 2) weighted by effect sizes for the reference allele obtained from the respective GWAS, and subsequently *z*-standardized. The first four multi-dimensional scaling (MDS) components calculated on the identity-by-state matrix of the genotype data in PLINK were used to adjust for population substructure.

#### Statistical analyses

Cognitive performance was analyzed aggregating all ten cognitive measures (multivariate analyses), as well as for each measure separately (univariate analyses).

General linear models were used for multivariate analyses. A base model (multivariate model 1) was constructed with CTQ sum as a predictor and all cognitive measures as dependent variables, while controlling for age and sex (as nuisance variables). In multivariate model 2, MDD diagnosis, PE, and PGS for MDD and EdA were then simultaneously added as predictors to this base model in order to control for all potentially confounding variables. This inclusive statistical approach provides a conservative estimate of the association between CM and cognition, because each additional control variable decreases the independent predictive value of CM (even for variables that may be completely independent of CM due to unsystematic error variance that can co-vary with CM).

Potential interaction effects of CTQ sum with age, sex, MDD diagnosis, PE, and both PGS variables were additionally tested in exploratory multivariate models. Multivariate models in which each potentially confounding variable is included separately (in order to delineate the role of each for the association between maltreatment and cognition), and analyses regarding the influence of current depression severity and medication to the above models are reported in the Supplementary. Multivariate partial eta squared (*η*²_p_) was used as a measure of effect size for all multivariate analyses.

Exploratory univariate follow-up analyses for each cognitive measure were subsequently conducted using multiple regression models for differentiation of domain-specific associations. Again, a base model was constructed with CTQ sum as a predictor, controlling for age and sex (univariate model 1) for each cognitive measure respectively. MDD diagnosis, PE, and PGS for MDD and EdA were then added separately to this base model resulting in univariate models 2, 3, 4, and 5, as well as all combined in model 6 (Table 2). This allows for an estimation of the role of these variables in the relationship between CM and cognitive abilities relative to each other and combined. Due to the large number of statistical tests in these analyses, an FDR-corrected significance threshold (*q* = 0.05) was applied across all 140 significance tests including variables of interest (including effects of CTQ sum, MDD diagnosis, PE and both PGS, while excluding nuisance variables age and sex from the correction) in all univariate models following the Benjamini–Hochberg procedure [38].

**Table 2:** Comparison of multivariable regression models predicting univariate cognitive performance.

	Model 1—base model					Model 2—MDD diagnosis					Model 3—PE					Model 4—MDD PGS					Model 5—EdA PGS					Model 6—all covariates				
	*B*	SE B	Beta	*P*	*R*²	*B*	SE B	Beta	*P*	*R*²	*B*	SE B	Beta	*P*	*R*²	*B*	SE B	Beta	*P*	*R*²	*B*	SE B	Beta	*P*	*R*²	*B*	SE B	Beta	*P*	*R*²
Corsi A																														
Age	−0.046	0.004	−0.309	<0.001		−0.045	0.004	−0.301	<0.001		−0.043	0.004	−0.287	<0.001		−0.046	0.004	−0.309	<0.001		−0.046	0.004	−0.308	<0.001		−0.042	0.004	−0.283	<0.001	
Sex	−0.152	0.109	−0.038	0.163		−0.166	0.109	−0.041	0.127		−0.164	0.109	−0.040	0.132		−0.149	0.109	−0.037	0.171		−0.159	0.109	−0.039	0.145		−0.176	0.108	−0.043	0.106	
CTQ sum	−0.016	0.004	−0.116	<0.001		−0.010	0.004	−0.070	0.024		−0.014	0.004	−0.101	<0.001		−0.015	0.004	−0.110	<0.001		−0.016	0.004	−0.112	<0.001		−0.007	0.004	−0.053	0.092	
MDD						−0.385	0.122	−0.098	0.002																	−0.350	0.122	−0.089	0.004	
PE											0.049	0.019	0.077	0.008												0.040	0.019	0.063	0.032	
MDD PGS																−0.149	0.053	−0.076	0.005							−0.132	0.053	−0.067	0.013	
EdA PGS																					0.060	0.053	0.031	0.258		0.039	0.054	0.020	0.464	
*Model*					0.124					0.130					0.128					0.129					0.124					0.137
Corsi B																														
Age	−0.048	0.004	−0.338	<0.001		−0.047	0.004	−0.329	<0.001		−0.044	0.004	−0.306	<0.001		−0.048	0.004	−0.338	<0.001		−0.048	0.004	−0.337	<0.001		−0.043	0.004	−0.301	<0.001	
Sex	−0.133	0.101	−0.034	0.189		−0.148	0.101	−0.038	0.143		−0.150	0.101	−0.039	0.138		−0.130	0.101	−0.034	0.199		−0.141	0.102	−0.036	0.166		−0.162	0.101	−0.042	0.106	
CTQ sum	−0.023	0.004	−0.174	<0.001		−0.016	0.004	−0.121	<0.001		−0.020	0.004	−0.151	<0.001		−0.022	0.004	−0.168	<0.001		−0.022	0.004	−0.170	<0.001		−0.013	0.004	−0.099	0.001	
MDD						−0.422	0.113	−0.112	<0.001																	−0.379	0.113	−0.101	0.001	
PE											0.069	0.017	0.112	<0.001												0.060	0.017	0.098	0.001	
MDD PGS																−0.136	0.049	−0.072	0.006							−0.115	0.049	−0.061	0.020	
EdA PGS																					0.065	0.050	0.035	0.188		0.037	0.050	0.019	0.463	
*Model*					0.169					0.178					0.179					0.174					0.170					0.189
LNST																														
Age	−0.044	0.007	−0.178	<0.001		−0.043	0.007	−0.172	<0.001		−0.038	0.007	−0.154	<0.001		−0.044	0.007	−0.177	<0.001		−0.043	0.007	−0.174	<0.001		−0.037	0.007	−0.151	<0.001	
Sex	0.020	0.187	0.003	0.914		0.004	0.187	0.001	0.981		0 < 0.001	0.186	0.000	0.999		0.020	0.187	0.003	0.914		−0.013	0.186	−0.002	0.946		−0.042	0.186	−0.006	0.823	
CTQ sum	−0.036	0.007	−0.155	<0.001		−0.028	0.007	−0.121	<0.001		−0.032	0.007	−0.138	<0.001		−0.036	0.007	−0.155	<0.001		−0.033	0.007	−0.144	<0.001		−0.023	0.007	−0.100	0.002	
MDD						−0.466	0.209	−0.071	0.026																	−0.442	0.209	−0.068	0.034	
PE											0.088	0.032	0.084	0.005												0.070	0.032	0.066	0.029	
MDD PGS																0.000	0.091	0.000	0.998							0.032	0.091	0.010	0.722	
EdA PGS																					0.291	0.091	0.089	0.001		0.263	0.092	0.081	0.004	
*Model*					0.065					0.068					0.070					0.064					0.072					0.077
TMT A–B																														
Age	−0.328	0.042	−0.220	<0.001		−0.317	0.042	−0.212	<0.001		−0.283	0.044	−0.189	<0.001		−0.328	0.042	−0.220	<0.001		−0.326	0.042	−0.218	<0.001		−0.275	0.044	−0.184	<0.001	
Sex	1.559	1.117	0.039	0.163		1.436	1.114	0.036	0.198		1.393	1.113	0.034	0.211		1.553	1.117	0.038	0.165		1.443	1.117	0.036	0.197		1.193	1.111	0.029	0.283	
CTQ sum	−0.193	0.039	−0.139	<0.001		−0.132	0.044	−0.095	0.003		−0.163	0.040	−0.118	<0.001		−0.194	0.039	−0.141	<0.001		−0.183	0.039	−0.133	<0.001		−0.104	0.045	−0.075	0.020	
MDD						−3.674	1.249	−0.093	0.003																	−3.465	1.249	−0.088	0.006	
PE											0.680	0.190	0.107	<0.001												0.607	0.192	0.095	0.002	
MDD PGS																0.316	0.546	0.016	0.562							0.542	0.544	0.027	0.319	
EdA PGS																					1.070	0.547	0.054	0.051		0.837	0.551	0.043	0.129	
*Model*					0.079					0.085					0.088					0.079					0.082					0.094
TMT A																														
Age	−0.263	0.020	−0.356	<0.001		−0.258	0.020	−0.349	<0.001		−0.247	0.021	−0.335	<0.001		−0.263	0.020	−0.356	<0.001		−0.263	0.020	−0.356	<0.001		−0.244	0.021	−0.331	<0.001	
Sex	0.603	0.533	0.030	0.258		0.544	0.532	0.027	0.306		0.548	0.532	0.027	0.303		0.612	0.533	0.031	0.251		0.594	0.534	0.030	0.266		0.509	0.532	0.025	0.339	
CTQ sum	−0.052	0.019	−0.076	0.005		−0.024	0.021	−0.035	0.251		−0.042	0.019	−0.061	0.028		−0.050	0.019	−0.073	0.008		−0.051	0.019	−0.075	0.006		−0.016	0.021	−0.023	0.464	
MDD						−1.678	0.596	−0.086	0.005																	−1.538	0.598	−0.079	0.010	
PE											0.230	0.091	0.073	0.012												0.207	0.092	0.066	0.025	
MDD PGS																−0.379	0.260	−0.039	0.146							−0.305	0.260	−0.031	0.240	
EdA PGS																					0.077	0.261	0.008	0.770		−0.019	0.263	−0.002	0.943	
*Model*					0.143					0.148					0.146					0.143					0.142					0.150
DSST																														
Age	−0.396	0.024	−0.410	<0.001		−0.380	0.024	−0.393	<0.001		−0.365	0.025	−0.378	<0.001		−0.396	0.024	−0.410	<0.001		−0.395	0.024	−0.409	<0.001		−0.354	0.024	−0.366	<0.001	
Sex	5.085	0.637	0.194	<0.001		4.898	0.622	0.187	<0.001		4.972	0.633	0.190	<0.001		5.096	0.637	0.194	<0.001		5.013	0.637	0.191	<0.001		4.766	0.620	0.182	<0.001	
CTQ sum	−0.193	0.022	−0.216	<0.001		−0.103	0.025	−0.115	<0.001		−0.173	0.023	−0.193	<0.001		−0.190	0.022	−0.213	<0.001		−0.188	0.022	−0.210	<0.001		−0.084	0.025	−0.094	0.001	
MDD						−5.429	0.698	−0.213	<0.001																	−5.224	0.697	−0.204	<0.001	
PE											0.469	0.108	0.113	<0.001												0.380	0.107	0.092	<0.001	
MDD PGS																−0.470	0.311	−0.037	0.131							−0.259	0.303	−0.020	0.394	
EdA PGS																					0.623	0.312	0.049	0.046		0.470	0.307	0.037	0.126	
*Model*					0.285					0.319					0.296					0.286					0.287					0.327
d2																														
Age	−1.466	0.087	−0.429	<0.001		−1.427	0.087	−0.417	<0.001		−1.353	0.090	−0.396	<0.001		−1.466	0.087	−0.429	<0.001		−1.457	0.087	−0.426	<0.001		−1.330	0.090	−0.389	<0.001	
Sex	4.936	2.311	0.053	0.033		4.528	2.291	0.049	0.048		4.512	2.297	0.049	0.050		4.980	2.309	0.054	0.031		4.495	2.304	0.049	0.051		3.868	2.274	0.042	0.089	
CTQ sum	−0.563	0.081	−0.178	<0.001		−0.354	0.091	−0.112	<0.001		−0.488	0.082	−0.154	<0.001		−0.551	0.081	−0.174	<0.001		−0.527	0.081	−0.166	<0.001		−0.267	0.092	−0.084	0.004	
MDD						−12.550	2.569	−0.139	<0.001																	−11.802	2.555	−0.131	<0.001	
PE											1.690	0.392	0.116	<0.001												1.349	0.394	0.092	0.001	
MDD PGS																−1.996	1.127	−0.044	0.077							−1.353	1.110	−0.030	0.223	
EdA PGS																					3.978	1.127	0.089	<0.001		3.380	1.125	0.075	0.003	
*Model*					0.248					0.262					0.259					0.249					0.255					0.276
*VLMT A*																														
Age	−0.266	0.019	−0.365	<0.001		−0.261	0.019	−0.358	<0.001		−0.242	0.020	−0.332	<0.001		−0.266	0.019	−0.365	<0.001		−0.264	0.019	−0.362	<0.001		−0.239	0.019	−0.328	<0.001	
Sex	4.394	0.500	0.221	<0.001		4.334	0.499	0.218	<0.001		4.310	0.497	0.217	<0.001		4.391	0.500	0.221	<0.001		4.294	0.499	0.216	<0.001		4.172	0.495	0.210	<0.001	
CTQ sum	−0.111	0.017	−0.164	<0.001		−0.083	0.020	−0.122	<0.001		−0.095	0.018	−0.141	<0.001		−0.112	0.018	−0.166	<0.001		−0.103	0.018	−0.153	<0.001		−0.066	0.020	−0.098	0.001	
MDD						−1.693	0.560	−0.088	0.003																	−1.604	0.557	−0.083	0.004	
PE											0.356	0.085	0.114	<0.001												0.304	0.086	0.097	<0.001	
MDD PGS																0.177	0.245	0.018	0.469							0.296	0.242	0.031	0.222	
EdA PGS																					0.862	0.244	0.089	<0.001		0.747	0.245	0.078	0.002	
*Model*					0.230					0.235					0.240					0.229					0.237					0.250
*VLMT B*																														
Age	−0.060	0.005	−0.311	<0.001		−0.059	0.005	−0.304	<0.001		−0.056	0.006	−0.290	<0.001		−0.060	0.005	−0.311	<0.001		−0.060	0.005	−0.309	<0.001		−0.056	0.006	−0.286	<0.001	
Sex	0.871	0.141	0.165	<0.001		0.855	0.141	0.162	<0.001		0.857	0.141	0.162	<0.001		0.872	0.141	0.165	<0.001		0.859	0.141	0.163	<0.001		0.836	0.141	0.158	<0.001	
CTQ sum	−0.013	0.005	−0.070	0.010		−0.006	0.006	−0.031	0.322		−0.010	0.005	−0.056	0.045		−0.012	0.005	−0.068	0.013		−0.012	0.005	−0.065	0.018		−0.003	0.006	−0.016	0.612	
MDD						−0.429	0.158	−0.083	0.007																	−0.402	0.159	−0.078	0.011	
PE											0.060	0.024	0.071	0.014												0.050	0.024	0.060	0.041	
MDD PGS																−0.056	0.069	−0.022	0.415							−0.035	0.069	−0.014	0.614	
EdA PGS																					0.099	0.069	0.039	0.151		0.078	0.070	0.030	0.263	
*Model*					0.135					0.139					0.138					0.134					0.135					0.142
*MWT−B*																														
Age	0.297	0.029	0.285	<0.001		0.301	0.029	0.290	<0.001		0.370	0.030	0.356	<0.001		0.296	0.029	0.285	<0.001		0.300	0.029	0.289	<0.001		0.372	0.030	0.358	<0.001	
Sex	0.074	0.777	0.003	0.925		0.026	0.777	0.001	0.973		−0.190	0.756	−0.007	0.802		0.063	0.777	0.002	0.936		−0.092	0.774	−0.003	0.905		−0.345	0.755	−0.012	0.647	
CTQ sum	−0.120	0.027	−0.125	<0.001		−0.097	0.031	−0.101	0.002		−0.072	0.027	−0.075	0.008		−0.123	0.027	−0.127	<0.001		−0.107	0.027	−0.112	<0.001		−0.053	0.030	−0.055	0.083	
MDD						−1.391	0.871	−0.051	0.111																	−0.996	0.848	−0.036	0.240	
PE											1.092	0.129	0.246	<0.001												1.042	0.131	0.234	<0.001	
MDD PGS																0.426	0.380	0.031	0.263							0.672	0.369	0.049	0.069	
EdA PGS																					1.431	0.379	0.105	<0.001		1.004	0.374	0.073	0.007	
*Model*					0.080					0.081					0.131					0.080					0.090					0.136

The variance explained by each model is presented with an adjusted *R*². Correction for multiple tests across all variables of interest (140 tests; all predictor variables except nuisance variables age and sex) following the Benjamini–Hochberg procedure [38] resulted in an FDR-corrected significance threshold of *P* < 0.036.

*MDD* major depressive disorder, *PE* parental education, *PGS* polygenic score, *EdA* educational attainment, *Corsi A* Corsi block tapping task forward, *Corsi B* Corsi block tapping task backward, *LNST* letter-number sequencing test, *TMT* trailmaking test (versions A and B), *DSST* digit symbol substitution test, *d2* d2 test, *VLMT* verbal learning and memory test (*VLMT A* short-term memory, *VLMT B* long-term memory), *MWT-B* multiple choice vocabulary test B.

Additional analyses regarding associations of cognition with specific types of CM (subscales of the CTQ) are provided in the Supplementary (Table S4).

Absence of multicollinearity and normal distribution of residuals of dependent variables are the two major statistical assumptions for using general linear models [39]. Due to low squared multiple correlations (SMCs) between all predictors (all *R*² < 0.282) and low variance inflation factors (all < 1.394) multicollinearity was not considered problematic for our analyses (Supplementary, Table S2) [39]. A more detailed examination of statistical assumptions (including distribution of the residuals of all dependent variables in Fig. S1) is presented in the Supplementary.

All statistical analyses were conducted using SPSS (IBM) version 25. An a priori significance threshold of *P* < 0.05 (*q* = 0.05 for FDR-corrected threshold) was defined.

## Results

### Multivariate analyses

In multivariate model 1 higher CTQ scores were significantly associated with lower multivariate cognitive performance (*F*_10,1187_ = 10.766, *P* < 0.001, *η*²_p_ = 0.083). Further, a significant interaction of CTQ sum with sex (*F*_10,1186_ = 2.085, *P* = 0.023, *η*²_p_ = 0.017) was found (post hoc tests across sex: males: *F*_10,437_ = 4.076, *P* < 0.001, *η*²_p_ = 0.085; females: *F*_10,739_ = 7.983, *P* < 0.001, *η*²_p_ = 0.097). The interaction of CTQ sum with age was not significant (*P* = 0.063).

**Fig. 1:**
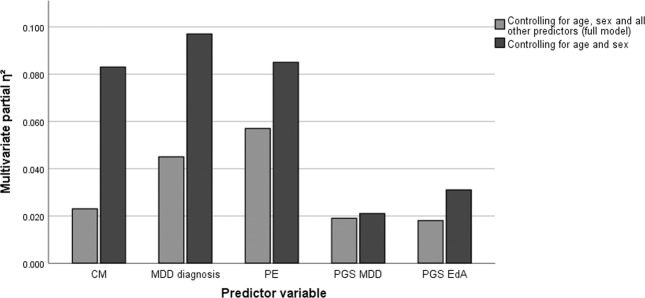
Effect size estimates for multivariate cognitive performance is presented over predictor variables. Variable effect sizes are compared in a reduced model with only one predictor while controlling for age and sex (dark gray), and in a full model controlling for age, sex, MDD diagnosis, PE, and polygenic scores for MDD and EdA (light gray). CM childhood maltreatment, MDD major depressive disorder, PE parental education, PGS polygenic score, EdA educational attainment.

Multivariate model 2 (including all variables) also yielded a significant effect of CTQ sum (*F*_10,1183_ = 2.763, *P* = 0.002, *η*²_p_ = 0.023). Further, significant effects were found for MDD diagnosis (*F*_10,1183_ = 5.537, *P* < 0.001, *η*²_p_ = 0.045), PE (*F*_10,1183_ = 7.178, *P* < 0.001, *η*²_p_ = 0.057), MDD PGS (*F*_10,1183_ = 2.267, *P* = 0.013, *η*²_p_ = 0.019), and EdA PGS (*F*_10,1183_ = 2.139, *P* = 0.019, *η*²_p_ = 0.018), all in the expected directions. A significant interaction of CTQ sum with sex (*F*_10,1182_ = 2.188, *P* = 0.016, *η*²_p_ = 0.018) and with age (*F*_10,1182_ = 1.977, *P* = 0.033, *η*²_p_ = 0.016) were found (post hoc tests per sex: males: *F*_10,433_ = 2.156, *P* = 0.019, *η*²_p_ = 0.047; females: *F*_10,735_ = 2.060, *P* = 0.025, *η*²_p_ = 0.027). No interaction effects of CTQ sum with MDD diagnosis, PE or either PGS was found (all *P* > 0.105). Multivariate results of models including each of the predictors separately (while controlling for age and sex) are presented in Fig. 1 and in further detail in Supplementary Table S3.

Adding MDS scores as covariates to any of the multivariate models did not qualitatively alter any statistical conclusions.

Male and female participants did not differ in age, PE, CTQ sum, either PGS, depression severity or most cognitive measures. Small but significant differences with higher scores in females were found in two of five maltreatment subscales (emotional and sexual abuse), but in no other subscales (Supplementary, Table S1). Further exploratory analyses yielded a significant CTQ sum × sex × diagnosis interaction, driven by a non-significant CTQ sum × sex interaction in HCs and a trend interaction between CTQ sum and sex in MDD participants (Supplementary and Fig. S3).

### Univariate follow-up analyses of separate cognitive measures

The FDR correction as described above, resulted in a corrected significance threshold of *P* < 0.036. The pattern of univariate results (Table 2) shows that when controlling for age and sex (base model), the relationship between CTQ sum and cognitive performance was significant for all cognitive measures in the expected direction (all *P* < 0.010) with a mean effect size of *β* = −0.098 (ranging from *β* = −0.070 for VLMT B to *β* = −0.216 for the DSST).

When MDD diagnosis was added to the base model the effects of CTQ sum on TMT A and VLMT B became non-significant (both *P* > 0.251) but was still significant for all other domains (all *P* < 0.024). Mean reduction in CTQ sum beta values in model 2 compared to model 1 was 36.24%, ranging from 19.27% (MWT-B) to 56.40% (VLMT B). A negative main effect of MDD diagnosis was significant for all cognitive measures (all *P* < 0.026) except the MWT-B (*P* = 0.111).

The negative relationship between CTQ sum and VLMT B was no longer significant when adding PE to the base model (*P* = 0.045). The association of CTQ sum with all other cognitive measures was still significant when adding PE (all *P* < 0.028). Mean standardized beta reductions of CTQ sum in the model with PE (model 3) compared to model 1 was 17.18%, ranging from 10.99% (LNST) to 40.20% (MWT-B). Further, results show that a positive main effect of PE was significant for all cognitive domains (all *P* < 0.014).

Adding MDD PGS in univariate model 4 did not qualitatively alter the effects of CTQ sum in any domain when compared to model 1. In model 4 CTQ sum was still a significant predictor for all univariate cognitive measures. Mean standardized beta reduction of CTQ sum from model 1 to model 4 was 1.58%. Model 4 further yielded a significant negative association of MDD PGS with Corsi A and Corsi B (both *P* < 0.006). No main effect of MDD PGS was found for any other domain (all *P* > 0.077).

EdA PGS was added as a predictor to the base model in univariate model 5 which did not alter CTQ effects substantially in any cognitive domain when comparing model 1 and model 5. The negative association between CTQ sum and cognitive ability was still significant for all cognitive measures (all *P* < 0.018). Mean standardized beta reduction in CTQ sum effects from model 1 to model 5 was 5.26% (ranging from 1.30% in TMT-A to 10.56% in MWT-B). Results further yielded a significant positive relationship between EdA PGS and LNST, d2, VLMT A, and the MWT-B (all *P* < 0.001), and a trend in the same direction for TMT A–B (*P* = 0.051). No significant effect of EdA PGS for any other cognitive measure was found (all *P* > 0.051).

In model 6 all variables are included. A negative association of CM with cognitive performance was still significant in this model for 6 out of the 10 cognitive measures (Corsi B, LNST, TMT A–B, DSST, d2, and VLMT A; all *P* < 0.020). Mean standardized beta reduction in CTQ sum effects from model 1 to model 6 was 53.18% (ranging from 35.25% in LNST to 77.33% in VLMT B).

## Discussion

We find that environmental (CM and PE), psychopathological (MDD diagnosis), and genetic variables (PGS for MDD and EdA) are all independently associated with cognitive functioning even when comprehensively controlling for each other. Our findings suggest that CM is associated with lower performance in a variety of cognitive domains. However, we show that a large portion of this association is explained particularly by MDD diagnosis and PE. These insights may aid an estimation of the potential impact of different loci of interventions for cognitive dysfunction.

In line with previous studies [10–12], we observed negative associations of CM with cognitive performance. Strongest CM-related deficits were found for working memory, sustained attention, and short-term memory. Weak but consistent associations between maltreatment and cognition were still present when statistically controlling for age, sex, MDD diagnosis, PE, and genetic predispositions for MDD and EdA. However, including MDD diagnosis and PE in our analyses revealed that both explained a substantial portion of the negative CM-cognition association, corroborating the notion that researchers are strongly advised to rigorously take these variables into account.

MDD, PE, and CM each show associations with cognitive dysfunction independent of each other. Further, we did not find that the CM association with cognition was moderated by MDD diagnosis or PE. Thus, the association of CM with cognitive ability was comparably pronounced across MDD and HC individuals, as well as across different parental educational contexts.

While our results suggest that CM is independently associated with cognition, an additional indirect influence of CM on cognitive ability may be mediated by an increased vulnerability to develop a MDD which in turn is associated with cognitive dysfunction [16]. Thus, the models that include MDD diagnoses factor out the additional impact of CM on cognition conveyed by a higher propensity for MDD.

Regarding the role of PE, our findings are in line with previous evidence that CM associations with cognition are still present when controlling for determinants of SES [11]. Danese et al. also report extensive associations of retrospectively and prospectively assessed CM with cognitive dysfunction in two large longitudinal cohorts, which however are drastically reduced or eliminated when controlling for SES and early age IQ of participants, thus questioning a causal link between CM and cognition [21]. In contrast, we still find this link even when controlling for PE and several other variables. Sample characteristics and differing assessment methods for CM, as well as differing determinants of SES may account for differences between studies and study subsamples. Agreement between retrospective and prospective measures of CM has been shown to be poor and both methodologies may be sensitive for different groups of subjects with maltreatment experiences [40]. Noteworthy, in a subsample that is comparable to our sample, regarding age at the time of assessment and type of CM assessment (retrospective self-reports), Danese et al. report results that are more similar to our findings, in that CM associations with cognition are reduced but not eliminated when controlling for SES [21]. In addition the assessment of SES differed from ours: while we used PE as a proxy for familial SES, Danese et al. operationalized SES as composite PE, income and occupation status in one cohort and as occupation status in a second cohort [21]. Although, these components of SES seem to be highly correlated [21], they may be differentially associated with adverse outcomes [41]. Further, we included a subsample of MDD subjects which may further account for differences in results. Together both studies underline the importance of controlling for the influence of SES determinants, and emphasize caution in the causal interpretation of CM associations with cognition.

In contrast, the inclusion of the PGS for MDD and EdA in our models did not alter the CM association with cognition. Polygenic variables did neither confound nor moderate this relationship. Interestingly, this was not due to a lacking association between PGS and cognitive measures per se. We found that a genetic predisposition for MDD was associated with lower performance in the visuospatial working memory domain. Further, the PGS for EdA was positively associated with measures of working memory, attention, short-term memory, and verbal intelligence. Effect sizes for both PGS were rather small, however comparable with genetic effect sizes regarding other complex phenotypes [42]. While the association between the EdA PGS and cognition has been documented previously [37], to our knowledge we are the first to report a relationship between a MDD PGS with phenotypical cognitive dysfunction. This finding raises the question if premorbid cognitive deficits may mediate the genetic propensity for MDD, or if the association between genetics and cognition is mediated by a higher vulnerability for depression. There is evidence for both causal pathways [43, 44], pointing to a potentially mutually dependent relationship. The missing CM by PGS interactions indicate, that the genetic predispositions for MDD and EdA seem to be associated with cognition independently from the environmental effect of CM. However, this conclusion is limited to the distinct PGS utilized in this study. Specific candidate genes or different PGS for different phenotypes may moderate associations of CM with cognition as previously suggested [1].

Despite the remaining CM associations with cognitive dysfunction when controlling for various variables, the question of *clinical* significance remains. Dichotomous statistical significance has been discussed intensively and controversially [45]. Particularly in light of large samples, effects may become statistically significant while effect sizes may be negligible regarding their clinical implications. Cognitive dysfunction in remitted MDD subjects of comparable effect sizes as we found for CM has been reported previously and such remission-state cognitive deficits have further been associated with difficulties in occupational reintegration [46]. This evidence supports the notion of clinical significance of our findings, suggesting that even small to medium-scale cognitive dysfunction can be related to relevant real-life outcomes. These considerations underline the importance to target cognitive deficits in populations with maltreatment experiences.

It has been discussed if sex differences in CM incidence rates or differing stress reactivity mechanisms as a function of sex could account for differing prevalence rates in affective disorders [47, 48]. While we find slightly higher reports of sexual and emotional abuse by women, the overall maltreatment load does not seem to differ in our sample. These results complement previous findings of prevalence estimates within a community sample that show similar results [49]. Further, we find evidence that sex seems to marginally moderate associations between CM and cognitive functioning, particularly in adults without a psychiatric condition. These findings point to a complex relationship between maltreatment, sex, psychopathology, and later adverse outcomes as cognitive functioning, and inform future studies that investigate sex differences in this field of research.

Overall, our results may implicate that a large portion of CM-associated cognitive dysfunction may be alleviated when effectively treating depression. Correspondingly, it is well known that MDD-related cognitive dysfunction seems to be at least partially reversible in the course of antidepressant treatment [50]. However, findings also suggest an association between cognitive dysfunction and inferior treatment response to pharmacotherapeutic and psychotherapeutic interventions in MDD [18], as well as lower treatment response for MDD cases with maltreatment experiences [3, 4]. Although initial evidence suggests the general effectivity of cognitive trainings in the treatment of MDD [51], little is known about the mechanisms of the relationship between cognitive dysfunction and treatment outcome in patient groups that have experienced CM. Future studies should investigate the benefit of customized interventions that take into account or directly target cognitive deficits for this patient group.

A clear limitation of our findings is the cross-sectional nature of the data. Although intuitive, inferences about a causal link between CM and cognitive functioning remain speculative. A possible alternative causal pathway could originate from a third variable that equally affects CM and cognition. Such a third variable could be embodied by one of the candidate variables under investigation in the current study (i.e., PE or MDD diagnosis) or by other possibly unknown variables. Another limitation is, that CM was assessed using retrospective self-reports which may be biased by state psychiatric condition or memory effects [40]. While we did control for psychiatric condition, memory bias may limit the validity of our findings. Although this would suggest an increase of noise in the CM assessment there is no reason to assume this additional error variance to be systematic, thus not posing a fundamental limitation to our conclusions. Further, it is noteworthy that CM-cognition effects that we often refer to as dysfunctional may include adaptive components, yielding benefits to deal with hostile environments [52].

In summary, our study provides insights in individual portions of variance in cognitive dysfunction associated with CM, MDD, PE, and genetic predisposition. Our findings may have implications about the potential scope of effects of interventions addressing these variables, and add to the understanding of their complex interplay. It remains to be investigated if this knowledge can be translated into tangible benefits of customized therapeutic approaches that take into account individual cognitive profiles in the sense of personalized medicine.

## Funding and disclosure

This work is part of the German multicenter consortium “Neurobiology of Affective Disorders. A translational perspective on brain structure and function“, funded by the German Research Foundation (Deutsche Forschungsgemeinschaft DFG; Forschungsgruppe/Research Unit FOR2107). Principal investigators (PIs) with respective areas of responsibility and funding in the FOR2107 consortium are: Work Package WP1, FOR2107/MACS cohort and brainimaging: TK (speaker FOR2107; DFG grant numbers KI 588/14-1, KI 588/14-2), UD (co-speaker FOR2107; DA 1151/5-1, DA 1151/5-2), AK (KR 3822/5-1, KR 3822/7-2), IN (NE 2254/1-2), Carsten Konrad (KO 4291/3-1). WP5, genetics: MRietschel (RI 908/11-1, RI 908/11-2), MN (NO 246/10-1, NO 246/10-2), SW (WI 3439/3-1, WI 3439/3-2). WP6, multi-method data analytics: AJ (JA 1890/7-1, JA 1890/7-2), Tim Hahn (HA 7070/2-2), Bertram Müller-Myhsok (MU1315/8-2), Astrid Dempfle (DE 1614/3-1, DE 1614/3-2). CP1, biobank: Petra Pfefferle (PF 784/1-1, PF 784/1-2), Harald Renz (RE 737/20-1, 737/20-2). CP2, administration. TK (KI 588/15-1, KI 588/17-1), UD (DA 1151/6-1), Carsten Konrad (KO 4291/4-1). Data access and responsibility: All PIs take responsibility for the integrity of the respective study data and their components. All authors and coauthors had full access to all study data. The FOR2107 cohort project (WP1) was approved by the Ethics Committees of the Medical Faculties, University of Marburg (AZ: 07/14) and University of Münster (AZ: 2014-422-b-S). Biomedical financial interests or potential competing interests: TK received unrestricted educational grants from Servier, Janssen, Recordati, Aristo, Otsuka, neuraxpharm. No further potential competing interests are declared by the authors. Open access funding provided by Projekt DEAL.

## Supplementary information

Supplementary material
