# Supplementary material for: Childhood maltreatment and cognitive functioning: the role of depression, parental education, and polygenic predisposition
Source: Neuropsychopharmacology. 2020 Aug 14;46(5):891–9. doi: 10.1038/s41386-020-00794-6 (PMC8115656; doi:10.1038/s41386-020-00794-6)
Supplement: Supplementary file 1 — Supplementary material [file 41386_2020_794_MOESM1_ESM.doc]

**Supplementary information**

The following supplementary information includes exclusion criteria and recruitment strategies of participants, operationalization of neurocognitive measures, examination of statistical assumptions of the statistical methods used, and complementary results. Further, additional figures and tables are provided as specified below.

**Supplementary methods**

**Supplementary results**

**Figure S1. Distribution of residuals of all dependent variables in the full multivariate model**

**Figure S2. Mediation models testing mediating effects of depression severity and parental education between childhood maltreatment and cognitive functioning**

**Figure S3. CTQ sum associations with cognitive performance across sex and diagnosis groups**

**Table S1. Study sample characteristics by sex groups**

**Table S2. Multicollinearity statistics for model predictors**

**Table S3. General linear models predicting multivariate cognitive performance**

**Table S4. Correlation matrix of childhood trauma questionnaire subscales and cognitive measures**

**Supplementary methods**

*Exclusion criteria and participant recruitment*

Exclusion criteria were any history of acute neurological condition (e.g., concussion, stroke, tumor, neuro-inflammatory diseases), medical condition (e.g., cancer, chronic inflammatory, autoimmune, or heart diseases), substance-related addictions, and insufficient German language proficiency.

Study advertisement and participant recruitment were done in psychiatric hospitals, outpatient therapeutic offices, public places, and newspapers. Study variables were assessed by the Departments of Psychiatry either at the University Hospital of Marburg or at the University Hospital of Münster.

*Neurocognitive measures*

Working memory was measured using the Corsi block tapping task in a forward (Corsi A) and a backward (Corsi B) version (1), and the letter-number sequencing subtest (LNST) of the Wechsler Adult Intelligence Scale (WAIS) (2). The Corsi task captures the visuospatial and the LNST the verbal capacity of the working memory. Further, the trailmaking test A (TMT A) and trailmaking test B (TMT B) were administered (3). Both TMT scores were reverse coded in order that higher scores indicate better performance (as being true for the rest of the neurocognitive measures). The difference of TMT A and TMT B (TMT A-B) was used as a measure of executive function thus correcting the TMT B performance for individual processing speed and thereby yielding a more pure indicator of executive function (4). The TMT A score together with the digit symbol substitution test (DSST) of the WAIS were used as an indicator of processing speed. Sustained attention was assessed using the d2 test (overall hits subtracted by the number of mistakes) (5). Declarative episodic memory was assessed using the verbal learning and memory test (VLMT) (6), which is the German adaptation of the Rey Auditory Verbal Learning Test (RAVLT) (7). Following the procedure described by Helmstaedter and colleagues, declarative short term memory was assessed using the sum of items recalled correctly after each of five presentations of a word list (VLMT A), and the 30-minutes delayed recognition task of the VLMT was used as an indicator of declarative long term memory (VLMT B; corrected for false-positives) (8).

Further, the MWT-B (9) was administered – a German multiple choice vocabulary test that is mostly used as an indicator for verbal intelligence because of the verbal nature of the task items. However, moderate correlations have been found with general intelligence as measured by the WAIS (10). The score of the MWT-B was directly translated to IQ-points for analyses (9). Notably, despite high correlations with other measures of intelligence as the WAIS the MWT-B tends to overestimate individual IQ on average by approximately 16 points (10). However, since absolute IQ scores were not interpreted in the current study this should be negligible.

*Examination of statistical assumptions*

Absence of multicollinearity, and normal distribution of residuals of dependent variables are the most prominent assumptions of general linear models.

As covariates were expected to correlate, the extent of multicollinearity was assessed using squared multiple correlations (SMC). Thus, each predictor variable (covariates and fixed factors) was predicted by respective other covariates and fixed factors in multiple linear regression models. With all SMCs *R²*<.282 and all variance inflation factors <1.394 the extent of multicollinearity was not considered to be problematic (11).

Residuals of the multivariate models were plotted and visually compared to a normality distribution. Residuals of most dependent variables were fairly normally distributed. Strongest deviations from normality were observed for both TMT measures, as well as the VLMT B (all skewed right and leptokurtic). Exemplary plots of residuals for the full model, including age, sex, childhood trauma questionnaire (CTQ) sum, diagnosis, parental education (PE), and polygenic scores (PGS) for major depressive disorder (MDD) and educational attainment (EdA) as predictors are shown in Figure S1. Due to the large sample size used in the present analyses we expect our statistical approach to be robust to these deviations of normality (11).

**Supplementary results**

*Multivariate models with successive addition of predictor variables*

Table S1 presents results of multivariate models. Model results are compared between the full model including all predictor variables of interest (CTQ sum, MDD diagnosis, PE, PGS MDD and PGS EdA), as well as age and sex as control variables, with models only including each variable of interest separately (while controlling for age and sex).

*The role of current depression severity and medication*

In order to assess the influence of current depression severity on the relationship between CM and cognitive functioning, Hamilton Depression Rating Scale (HDRS)(12) scores were added to the multivariate models. Adding HDRS scores to the base model predicting multivariate cognitive performance with CTQ while controlling for age and sex (multivariate model 1) yielded a significant main effect of CTQ sum in the expected direction (*F*_10,1181_=4.533, *P<*.001, η²_p_=.037), and a significant main effect of HDRS (*F*_10,1181_=5.388, *P<*.001, η²_p_=.044) driven by a positive association between depression severity and cognitive functioning. No interaction between CTQ sum and HDRS score was found (*P*=.914).

In a subsequent step HDRS was added to multivariate model 2, resulting in a model including CTQ sum, MDD diagnosis, PE, MDD PGS, EdA PGS, HDRS score, age, and sex to predict multivariate cognitive functioning. This model yielded significant main effects of CTQ sum (*F*_10,1177_=2.167, *P*=.018, η²_p_=.018), MDD diagnosis (*F*_10,1177_=2.361, *P*=.009, η²_p_=.020), PE (*F*_10,1177_=6.990, *P<*.001, η²_p_=.056), MDD PGS (*F*_10,1177_=2.278, *P=*.012, η²_p_=.019), EdA PGS (*F*_10,1177_=1.999, *P=*.030, η²_p_=.017), and a non-significant trend for HDRS score (*F*_10,1177_=1.691, *P=*.078). Adding an interaction term between CTQ sum and HDRS score yielded a non-significant interaction effect (*P*=.952).

In order to investigate the influence of current medication on our analyses, a medication index was calculated from self-reports of type and dose of current psychopharmacologic treatment (described in previous publications) (13,14). Adding the medication index to multivariate model 1 yielded a significant main effect of CTQ sum (*F*_10,1186_=6.934, *P<*.001, η²_p_=.055), and a significant main effect of the medication index (*F*_10,1186_=6.612, *P<*.001, η²_p_=.053) both driven by negative associations with cognitive measures. Adding an interaction term between CTQ sum and the medication index yielded a non-significant interaction effect (*P*=.550), indicating that the CTQ sum association with cognition was independent of current psychopharmacological treatment.

When the medication index was added to multivariate model 2, significant main effects of CTQ sum (*F*_10,1182_=2.697, *P*=.003, η²_p_=.022), MDD diagnosis (*F*_10,1182_=2.610, *P*=.004, η²_p_=.022), PE (*F*_10,1182_=7.174, *P<*.001, η²_p_=.057), MDD PGS (*F*_10,1182_=2.278, *P=*.012, η²_p_=.019), EdA PGS (*F*_10,1182_=2.159, *P=*.018, η²_p_=.018), and a significant main effect for the medication index (*F*_10,1182_=3.436, *P<*.001, η²_p_=.028) emerged. Adding an interaction term between CTQ sum and the medication index yielded a non-significant interaction effect (*P*=.854).

In summary neither the inclusion of current depression severity, nor the inclusion of medication load in statistical models altered our results regarding the relationship between CM and cognition.

*Maltreatment subtypes*

In regard of the maltreatment subtype analysis, the pattern of results indicate that negative bivariate associations of CM with cognition are present similarly for all maltreatment subscales (Table S2). On average small effects sizes were found (emotional abuse: r=-.144; physical abuse: r=-.166; sexual abuse: r=-.096; emotional neglect: r=-.182; physical neglect=-.187). Descriptively smallest effect sizes were found for the sexual abuse subscale.

*Mediation analyses of parental education and depression severity*

Mediation analyses were conducted using PROCESS (15) to formally test a mediating effect of either MDD severity or parental education between CM and cognitive functioning. Importantly, this mediation analysis does not allow any conclusions in regard to causality due to the cross-sectional design of the study. Significance on a p<.05 level was determined using bootstrapping (N=5000 samples). For the mediation analyses a compound score for cognitive functioning was calculated (mean of all ten z-standardized cognitive measures). Further, the BDI score was used as a measure of the depression severity instead of utilizing the categorical MDD diagnosis variable in order to obtain a continuous variable. A partial mediation of MDD severity between CM and cognitive functioning was found with a significant indirect effect that took up 40.82% of the total effect. A partial mediation effect to a lesser extent was also found for PE with a significant indirect effect that took up 16% of the total effect. Results of the mediation models are presented in Figure S2.

*Interaction between maltreatment, sex and diagnosis*

An exploratory investigation of a three-way interaction between CTQ sum, sex and diagnosis was tested in a multivariate model predicting all ten cognitive measures (statistic estimates based on Pillai’s Lambda in order to provide robust results (11)). Main effects of CTQ sum, sex, age and diagnosis were included in the model, as well as the three-way interaction effect. The model yielded a significant three-way interaction (*F*_30,3555_=1.630, *P*=.017, η²_p_=.014). Additional post hoc tests revealed that the three-way interaction was driven by a non-significant interaction between CTQ sum and sex in healthy controls (*F*_10,649_=0.844, *P*=.586) but a trend interaction between CTQ sum and sex in MDD participants (*F*_10,523_=1.692, *P*=.079, η²_p_=.031). The three-way interaction is visualized in Figure S3.

**Figure S1. Distribution of residuals of all dependent variables in the full multivariate model.** Corsi A, Corsi block tapping task forward; Corsi B, Corsi block tapping task backward; LNST, letter-number sequencing test; TMT, trailmaking test (versions A and B); DSST, digit symbol substitution test; d2, d2 test; VLMT, verbal learning and memory test (VLMT A, short-term memory; VLMT B, long-term memory); MWT-B, multiple choice vocabulary test.

**Figure S2. Mediation models testing mediating effects of depression severity and parental education between childhood maltreatment and cognitive functioning.** Unstandardized coefficients are presented along with 95% confidence intervals (CI) and standard errors (SE). **P*<.05.


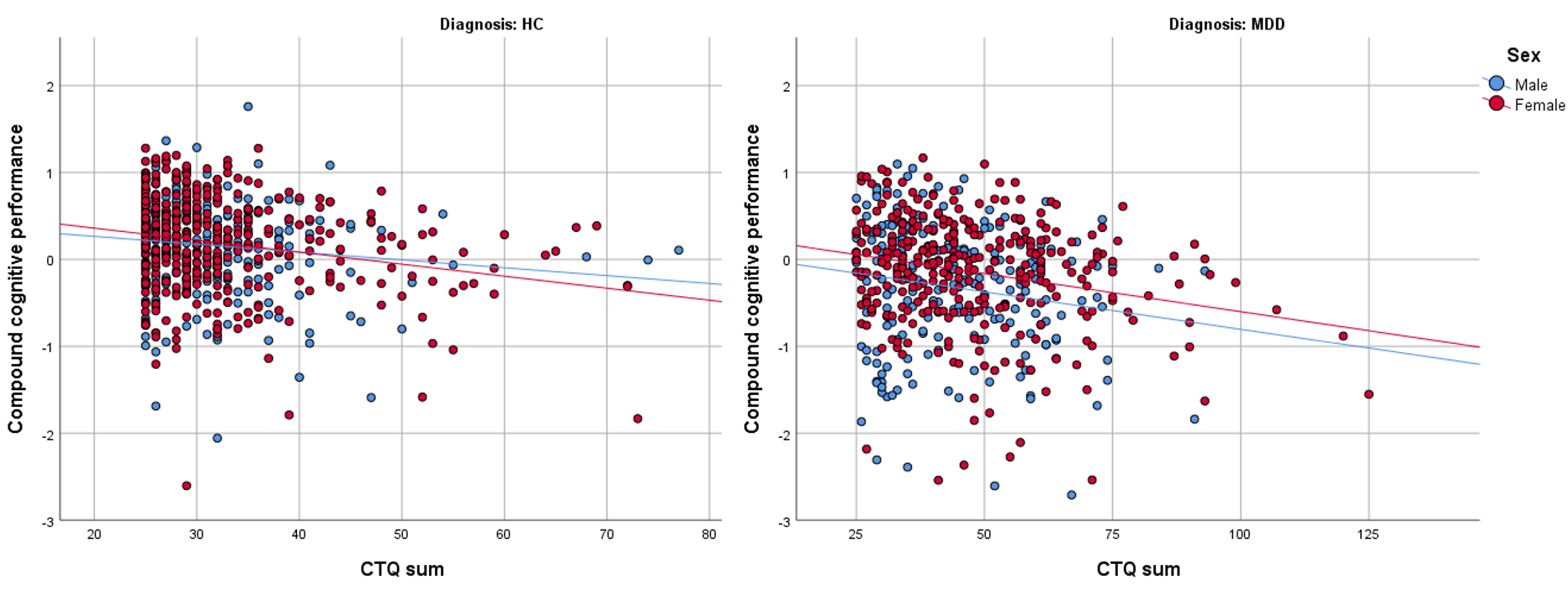


**Figure S3. CTQ sum associations with cognitive performance across sex and diagnosis groups.** Mean of z-standardized cognitive measures is provided as the compound cognitive performance. HC, healthy controls; MDD, major depression disorder.

**Table S1**

**Study sample characteristics by sex groups**

|  |  | Male | |  | Female | |  |
| --- | --- | --- | --- | --- | --- | --- | --- |
|  | N | Mean | SD |  | Mean | SD | p-Value |
| *Demographics* |  |  |  |  |  |  |  |
| Age | 1217 | 34.66 | 12.76 |  | 34.65 | 13.43 | .997 |
| PE | 1217 | 12.54 | 3.02 |  | 12.73 | 3.10 | .279 |
| *Maltreatment* |  |  |  |  |  |  |  |
| CTQ sum | 1217 | 37.83 | 12.78 |  | 38.78 | 14.97 | .242 |
| CTQ EA | 1217 | 8.55 | 4.27 |  | 9.10 | 4.80 | .038 |
| CTQ PA | 1217 | 6.14 | 2.36 |  | 6.29 | 2.79 | .321 |
| CTQ SA | 1217 | 5.28 | 1.44 |  | 6.00 | 2.74 | <.001 |
| CTQ EN | 1217 | 10.77 | 4.98 |  | 10.43 | 5.17 | .251 |
| CTQ PN | 1217 | 7.09 | 2.57 |  | 6.96 | 2.78 | .428 |
| *Cognitive measures* |  |  |  |  |  |  |  |
| Corsi A | 1214 | 9.11 | 2.10 |  | 8.95 | 1.87 | .164 |
| Corsi B | 1214 | 8.61 | 1.89 |  | 8.45 | 1.87 | .167 |
| LNST | 1212 | 16.27 | 3.52 |  | 16.25 | 3.08 | .931 |
| TMT A-B | 1210 | -29.56 | 21.00 |  | -28.22 | 18.71 | .260 |
| TMT A | 1214 | -24.91 | 10.25 |  | -24.35 | 9.37 | .342 |
| DSST | 1215 | 57.34 | 12.37 |  | 62.25 | 12.57 | <.001 |
| d2 | 1211 | 177.29 | 44.51 |  | 181.78 | 45.03 | .091 |
| VLMT A | 1215 | 54.86 | 10.32 |  | 59.14 | 8.79 | <.001 |
| VLMT B | 1214 | 12.93 | 3.07 |  | 13.79 | 2.14 | <.001 |
| MWT-B | 1214 | 113.50 | 14.10 |  | 113.45 | 13.40 | .955 |
| *Polygenic scores* |  |  |  |  |  |  |  |
| MDD PGS | 1217 | -0.02 | 1.08 |  | 0.01 | 0.94 | .662 |
| EdA PGS | 1217 | -0.06 | 0.95 |  | 0.04 | 1.03 | .070 |
| *Depression severity* |  |  |  |  |  |  |  |
| HDRS | 1212 | 5.28 | 6.80 |  | 5.15 | 6.73 | .752 |

*Note.* T-tests were used to test for significance of group differences. Statistics are presented assuming unequal variances in groups as most dependent variables produced a significant Levene’s test. Both PGS’ are presented z-standardized. For all cognitive measures, higher scores represent better performance. MDD, major depressive disorder; HC, healthy controls; f, female; m, male; CTQ, childhood trauma questionnaire; EA, emotional abuse; PA, physical abuse; SA, sexual abuse; EN, emotional neglect; PN, physical neglect; PE, parental education; Corsi A, Corsi block tapping task forward; Corsi B, Corsi block tapping task backward; LNST, letter-number sequencing test; TMT, trailmaking test (versions A and B); DSST, digit symbol substitution test; d2, d2 test; VLMT, verbal learning and memory test (VLMT A, short-term memory; VLMT B, long-term memory); MWT-B, multiple choice vocabulary test B; PGS, polygenic score; HDRS, Hamilton Depression Rating Scale.

**Table S2**

Multicollinearity statistics for model predictors

|  | SMC | VIF |
| --- | --- | --- |
| Age | 0.129 | 1.148 |
| Sex | 0.007 | 1.007 |
| CTQ sum | 0.282 | 1.393 |
| MDD | 0.255 | 1.342 |
| PE | 0.176 | 1.214 |
| MDD PGS | 0.015 | 1.015 |
| EdA PGS | 0.045 | 1.047 |

*Note.* SMC, squared multiple correlations; VIF, variance inflation factor; CTQ, childhood

trauma questionnaire; MDD, major depressive disorder; PE, parental education; PGS,

polygenic score; EdA, educational attainment.

**Table S3**

General linear models predicting multivariate cognitive performance

|  | Full model | |  | CM model | |  | MDD Model | |  | PE model | |  | PGS MDD model | |  | PGS EdA model | |
| --- | --- | --- | --- | --- | --- | --- | --- | --- | --- | --- | --- | --- | --- | --- | --- | --- | --- |
|  | *F* | η*²* |  | *F* | η*²* |  | *F* | η*²* |  | *F* | η*²* |  | *F* | η*²* |  | *F* | η*²* |
| Age | 73.619 | .384*** |  | 79.596 | .401*** |  | 81.266 | .406*** |  | 77.575 | .395*** |  | 88.295 | .427*** |  | 87.452 | .424*** |
| Sex | 17.618 | .130*** |  | 18.192 | .133*** |  | 17.785 | .130*** |  | 17.420 | .128*** |  | 17.515 | .129*** |  | 17.322 | .127*** |
| CTQ sum | 2.763 | .023** |  | 10.766 | .083*** |  |  |  |  |  |  |  |  |  |  |  |  |
| MDD diagnosis | 5.537 | .045*** |  |  |  |  | 12.800 | .097*** |  |  |  |  |  |  |  |  |  |
| PE | 7.178 | .057*** |  |  |  |  |  |  |  | 11.059 | .085*** |  |  |  |  |  |  |
| PGS MDD | 2.267 | .019* |  |  |  |  |  |  |  |  |  |  | 2.510 | .021** |  |  |  |
| PGS EdA | 2.139 | .018* |  |  |  |  |  |  |  |  |  |  |  |  |  | 3.859 | .031*** |

*Note.* A model with all predictor variables (full model) is compared to models that only include one of the predictor variables while controlling for age and sex. *F* is presented as the statistical parameter and partial η*²* is presented as a measure of effect size. CM, childhood maltreatment; MDD, major depressive disorder; PE, parental education; PGS, polygenic score; EdA, educational attainment; **P*<.05; ***P*<.01; ****P*<.001

**Table S4**

Correlation matrix of childhood trauma questionnaire subscales and cognitive measures

|  |  | CTQ sum | CTQ EA | CTQ PA | CTQ SA | CTQ EN | CTQ PN |
| --- | --- | --- | --- | --- | --- | --- | --- |
| Corsi A | | -.183*** | -.137*** | -.174*** | -.093** | -.162*** | -.167*** |
| Corsi B | | -.247*** | -.209*** | -.217*** | -.105*** | -.227*** | -.206*** |
| LNST | | -.192*** | -.152*** | -.157*** | -.107*** | -.164*** | -.191*** |
| TMT A-B | | -.184*** | -.130*** | -.171*** | -.088** | -.167*** | -.185*** |
| TMT A | | -.150*** | -.103*** | -.125*** | -.126*** | -.138*** | -.121*** |
| DSST | | -.296*** | -.216*** | -.239*** | -.149*** | -.302*** | -.249*** |
| d2 | | -.266*** | -.185*** | -.212*** | -.163*** | -.268*** | -.226*** |
| VLMT A | | -.233*** | -.159*** | -.169*** | -.097** | -.239*** | -.252*** |
| VLMT B | | -.130*** | -.098** | -.107*** | -.014 | -.131*** | -.149*** |
| MWT-B | | -.065* | -.046 | -.093** | -.014 | -.017 | -.127*** |

*Note.* Bivariate Pearson correlation coefficients are presented. CTQ, childhood trauma questionnaire; EA, emotional abuse; PA, physical abuse; SA, sexual abuse; EN, emotional neglect; PN, physical neglect; Corsi A, Corsi block tapping task forward; Corsi B, Corsi block tapping task backward; LNST, letter-number sequencing test; TMT, trailmaking test (versions A and B); DSST, digit symbol substitution test; d2, d2 test; VLMT, verbal learning and memory test (VLMT A, short-term memory; VLMT B, long-term memory); MWT-B, multiple choice vocabulary test B. *P<.05, **P<.01, ***P<.001.

**Supplementary references**

1. Corsi PM (1972): *Human Memory and the Medial Temporal Region of the Brain.* McGill University Montreal.

2. Wechsler D (1997): *WAIS-III Administration and Scoring Manual*. San Antonie, TX: The Psychological Corporation.

3. Reitan RM (1958): Validity of the trail making test as an indicator of organic brain damage. *Percept Mot Skills* 8: 271–276.

4. Sánchez-Cubillo I, Periáñez JA, Adrover-Roig D, Rodríguez-Sánchez JM, Ríos-Lago M, Tirapu J, Barceló F (2009): Construct validity of the Trail Making Test: Role of task-switching, working memory, inhibition/interference control, and visuomotor abilities. *J Int Neuropsychol Soc* 15: 438–450.

5. Brickenkamp R, Schmidt-Atzert L, Liepmann D (2010): *d2-R: Test d2 - Revision*, 1st ed. Göttingen: Hogrefe.

6. Helmstaedter C, Durwen HF (1990): VLMT: Verbaler Lern- und Merkfähigkeitstest. *Schweizer Arch für Neurol und Psychiatr* 141: 21–30.

7. Rey A (1964): *L’edamen Clinique En Psychologie*. Paris: Presses universitaires de France.

8. Helmstaedter C, Lendt M, Lux S (2001): *VLMT - Verbaler Lern- Und Merkfähigkeitstest*. Göttingen: Beltz Test GmbH.

9. Lehrl S (2005): *Mehrfachwahl-Wortschatz-Intelligenztest MWT-B*, 5th ed. Balingen: Spitta-Verlag.

10. Satzger W, Fessmann H, Engel RR (2002): Liefern HAWIE-R, WST und MWT-B vergleichbare IQ-Werte? *Zeitschrift für Differ und Diagnostische Psychol* 23: 159–170.

11. Tabachnick BG, Fidell LS (2007): *Using Multivariate Statistics*, 5th ed. ((S. Hartmann & T. Felser, editors)). Boston: Pearson.

12. Hamilton M (1960): A Rating Scale for depression. *J Neurol Neurosurg Psychiatry* 23: 56–62.

13. Zaremba D, Dohm K, Redlich R, Grotegerd D, Strojny R, Meinert S, *et al.* (2018): Association of Brain Cortical Changes With Relapse in Patients With Major Depressive Disorder. *JAMA Psychiatry* 75: 484–492.

14. Redlich R, Almeida JR, Grotegerd D, Opel N, Kugel H, Heindel W, *et al.* (2014): Brain Morphometric Biomarkers Distinguishing Unipolar and Bipolar Depression: A Voxel-Based Morphometry-Pattern Classification Approach. *JAMA Psychiatry* 71: 1222–1230.

15. Hayes AF (2017): *Introduction to Mediation, Moderation, and Conditional Process Analysis: A Regression-Based Approach*, 2nd ed. New York: Guilford Press.
